# Neuropeptide Control of Feeding Behavior in Birds and Its Difference with Mammals

**DOI:** 10.3389/fnins.2016.00485

**Published:** 2016-11-02

**Authors:** Tetsuya Tachibana, Kazuyoshi Tsutsui

**Affiliations:** ^1^Laboratory of Animal Production, Department of Agrobiological Science, Faculty of Agriculture, Ehime UniversityMatsuyama, Japan; ^2^Laboratory of Integrative Brain Sciences, Department of Biology and Center for Medical Life Science, Waseda UniversityTokyo, Japan

**Keywords:** feeding regulatory peptides, neuropeptides, feeding, central nervous system, birds

## Abstract

Feeding is an essential behavior for animals to sustain their lives. Over the past several decades, many neuropeptides that regulate feeding behavior have been identified in vertebrates. These neuropeptides are called “feeding regulatory neuropeptides.” There have been numerous studies on the role of feeding regulatory neuropeptides in vertebrates including birds. Some feeding regulatory neuropeptides show different effects on feeding behavior between birds and other vertebrates, particularly mammals. The difference is marked with orexigenic neuropeptides. For example, melanin-concentrating hormone, orexin, and motilin, which are regarded as orexigenic neuropeptides in mammals, have no effect on feeding behavior in birds. Furthermore, ghrelin and growth hormone-releasing hormone, which are also known as orexigenic neuropeptides in mammals, suppress feeding behavior in birds. Thus, it is likely that the feeding regulatory mechanism has changed during the evolution of vertebrates. This review summarizes the recent knowledge of peptidergic feeding regulatory factors in birds and discusses the difference in their action between birds and other vertebrates.

## Introduction

Domestic chickens (*Gallus gallus domesticus*) are raised worldwide for the production of meat and eggs as food for humans. Chickens have been genetically selected for efficient meat and egg production and thereby many strains have been developed. However, they are divided broadly into two strains: One is selected for meat production (meat-type chickens) and the other is for egg production (layer-type chickens) (Denbow, 1994). Meat-type strains including broilers have been selected for rapid early growth (Denbow, 1994). Nutrient and food intake during the chick stage is thought to be important for sustaining growth in meat-type chickens, thus it is essential to understanding the mechanisms underlying ingestion in these, and other, types of birds (Siegel and Wisman, 1966; Denbow, 1994).

Food intake is regulated by complex systems involving both central and peripheral sites of control, such as the gastrointestinal tract, liver, and brain. Food must enter the gastrointestinal tract, where it then must be digested and absorbed, and it is expected that this organ system has a role in regulating food intake. In most avian species, food is temporarily stored in the crop and then enters the proventriculus and gizzard. The expansion of the crop and gizzard is expected to contribute to the termination of short-term feeding (Savory, 1999). The liver is also important in regulating feeding behavior because injection of glucose into the portal vein suppresses feeding behavior in chickens (Denbow, 1994). Intraheptic injection of other nutrients, such as lipids and amino acids, also affects feeding behavior (Denbow, 1994). Peptidergic hormones such as cholecystokinin (CCK) are also thought to be related to the regulation of feeding behavior (Denbow, 1994). These peripheral factors are sent to the central nervous system via humoral and neural pathways (Denbow, 1994; Savory, 1999). The control of the beak and the visual, taste, and smell sensations are also thought to be important in controlling feeding behavior. These sensations are sent to the central nervous system via specific pathways, such as the trigeminal sensorimotor system (for the control of grasping and mandibulation of the diet), the tectofugal and thalamofugal pathways (for visual sensation), the gustatory system, and the olfactory pathway (Kuenzel, 1989). The autonomic nervous system/parasympathetic pathway is also important in regulating feeding behavior in birds (Kuenzel, 1989).

The central nervous system plays an important role in regulating feeding behavior in birds (Kuenzel, 1989, 1994). The hypothalamus is thought to have important roles in regulating feeding in birds. Classically, the ventromedial nucleus (VMN) and lateral hypothalamic area (LHA) are respectively known as satiety and feeding centers in birds as well as mammals. In addition to these nuclei, other hypothalamic nuclei, such as the paraventricular nucleus (PVN) and infundibular nucleus [IN, an avian homolog of the arcuate nucleus (ARC)], are thought to be involved in regulating feeding behavior.

Over the past several decades, numerous bioactive molecules have been identified as affecting the activity of the neural network related to regulating feeding behavior in birds. Among them, peptidergic molecules that act in the central nervous system are sometimes called “feeding regulatory neuropeptides” and are well-studied in birds (Furuse et al., 2007). Feeding regulatory neuropeptides are conveniently divided into two categories: One suppresses feeding behavior and are called anorexigenic or anorectic neuropeptides, and the other stimulates feeding behavior and are called orexigenic neuropeptides.

The roles of feeding regulatory neuropeptides have been well-studied not only in birds but also in other vertebrates, such as mammals (Kageyama et al., 2012), amphibians (Carr et al., 2002), and teleosts (Volkoff et al., 2005). In these studies, the effects of some feeding regulatory peptides in birds are somewhat different from the effects in other vertebrates, in particular mammals (Furuse et al., 2007). This review summarizes the recent knowledge of feeding regulatory peptides in birds and discusses the difference in their action between birds and other vertebrates. Most of the studies on avian feeding regulatory peptides are well-performed using neonatal chicks (Furuse et al., 2007; Bungo et al., 2011; Honda, 2016), so previous findings on chicks are mainly described.

## Peripheral peptidergic hormones

Several peptidergic hormones released from peripheral tissues are thought to affect feeding behavior. Among them, leptin, ghrelin, and CCK, which are mainly released from adipose tissue, stomach, and small intestine, respectively, are expected to play a key role in regulating feeding behavior in mammals (Friedman and Halaas, 1998; Miyasaka and Funakoshi, 2003; Kojima and Kangawa, 2005). These hormones have been identified in birds, but some of them produce different effects in birds compared to other vertebrates.

### Leptin

Leptin is the protein product of the *obese* gene and is released from adipose tissue (Friedman and Halaas, 1998). Central or peripheral injection of leptin induces a decline in food intake, enhancement of energy expenditure, and decrease in adipose tissue and body weight in mammals (Friedman and Halaas, 1998). In addition, because lacking normal leptin or a leptin receptor induces obesity in mice (Friedman and Halaas, 1998), leptin is thought to have an important role in regulating body fat volume and body weight. The anorexigenic effect of leptin has also been observed in teleosts. For example, intracerebroventricular (ICV) or intraperitoneal (IP) injection of mouse leptin suppresses feeding behavior in goldfish, in part by modulating the orexigenic effect of neuropeptide Y (NPY) and orexin (Volkoff et al., 2003). Furthermore, leptin receptor-deficient medaka showed hyperphagia, increase in body weight, and deposition of visceral fat (Chisada et al., 2014). These results suggest that leptin is an important anorexigenic peptide in vertebrates.

The anorexigenic effect of leptin has also been observed in Asian blue quail (Lõhmus et al., 2006) and wintering white-throated sparrows (Cerasale et al., 2011). Similar to these avian species, ICV injection of human leptin suppresses feeding behavior in young chickens (Denbow et al., 2000). However, the effect seems to depend on age: ICV injection of murine leptin has no effect on feeding in neonatal chicks (Bungo et al., 1999b). Thus, the role of leptin on feeding behavior in chicks is somewhat different from that in mammals.

Taouis et al. (1998) isolated the *leptin* gene in chickens, and the amino acid sequence of chicken leptin has high homology to mammalian counterparts. However, other researchers could not find the isolated *leptin* gene in the sequenced chicken genome (Friedman-Einat et al., 1999) or other avian genomes. Recently, the *leptin* gene has been isolated in several kinds of birds (Boswell and Dunn, 2015). The chicken *leptin* gene has also been newly identified (Seroussi et al., 2016). Avian leptin exhibits ~30% amino acid identity to its mouse and human counterparts (Boswell and Dunn, 2015). These findings suggest the necessity of reexamining the effect of leptin on feeding behavior in birds. Studies using these avian leptins are needed to clarify the actual role of leptin in regulating feeding in avian species.

### Ghrelin

Ghrelin, an acylated peptide hormone released from the stomach, was originally identified as an endogenous stimulator of growth hormone (GH) release from the anterior pituitary, but later work revealed that this peptide is related to regulating feeding in mammals. In rodents, central or peripheral injection of ghrelin increases feeding behavior (Nakazato et al., 2001), demonstrating that ghrelin is one orexigenic peptide in mammals.

Ghrelin has also been identified in non-mammalian species including chickens (Kaiya et al., 2002) and stimulates GH release (Kaiya et al., 2002). In addition, ghrelin was also demonstrated to stimulate feeding behavior in teleosts (Unniappan et al., 2002) and bullfrog larvae (Shimizu et al., 2014). Thus, ghrelin is regarded as an orexigenic peptide in both mammals and fish. In contrast, ICV injection of rat ghrelin inhibits rather than stimulates feeding behavior in neonatal chicks (Furuse et al., 2001). Moreover, chicken ghrelin suppresses feeding behavior in neonatal chicks when administered centrally (Saito et al., 2002a), demonstrating that central ghrelin is not an orexigenic peptide in chickens. ICV injection of rat ghrelin also suppresses feeding behavior in adult Japanese quail (Shousha et al., 2005a). Thus, the action of ghrelin in the feeding regulatory mechanism in the brain of birds is the opposite of that in mammals and fish.

ICV-injected ghrelin induces several behavioral changes such as vocalization and hyper activity in neonatal chicks (Saito et al., 2002b). These behaviors are also induced by ICV injection of corticotropin-releasing hormone (CRH) (Zhang et al., 2001a), and CRH itself is regarded as an anorexigenic peptide in chicks (Furuse et al., 1997b). These facts imply that the effect of central ghrelin is related to the CRH systems. Indeed, co-injection of astressin, a CRH receptor antagonist, restores the ghrelin-induced decrease in food intake in neonatal chicks (Saito et al., 2005). Thus, the anorexigenic effect of central ghrelin is likely to be mediated by the CRH system in chicks. Further studies revealed that the anorexigenic effect of ghrelin is also mediated by the serotonergic system (Zendehdel et al., 2013) and β-adrenergic system (Zendehdel and Hassanpour, 2014). In rodents, the orexigenic effect of ghrelin is thought to be mediated by NPY (Nakazato et al., 2001). In chicks, ICV injection of chicken ghrelin has no effect on the mRNA expression of NPY (Saito et al., 2005). Since ghrelin does not activate NPY neuron in the brain, ghrelin might lack its orexigenic effect in birds.

In contrast to central action, the peripheral action of ghrelin is not consistent in birds. IP injection of lower doses of rat ghrelin (0.4–0.9 nmol/100 g body weight) stimulates feeding behavior, whereas a higher dose (2.4 nmol/100 g body weight) suppresses feeding behavior in adult Japanese quail (Shousha et al., 2005a). In chicks, intravenous injection of chicken ghrelin slightly but significantly suppresses feeding behavior (Geelissen et al., 2006). On the other hand, intravenous injection of chicken ghrelin has no effect on food intake in neonatal chicks (Kaiya et al., 2007). Nevertheless, the change in plasma ghrelin concentration after food deprivation in chicks is similar to rats (Toshinai et al., 2001): 12-h food deprivation increases plasma ghrelin concentration and ghrelin content in the proventriculus in chicks (Kaiya et al., 2007). In rodents, the peripheral ghrelin signal, which is sent to the nucleus of the solitary tract via the vagus nerve, is transmitted to the hypothalamus via noradrenergic neurons, and thereafter activates NPY neurons (Date et al., 2006). It is possible that peripheral ghrelin does not affect this pathway in birds.

## Anorexigenic neuropeptides

Table 1 summarizes the representative candidates for anorexigenic neuropeptides in birds. These peptides are roughly categorized as the CRH family, melanocortin, glucagon superfamily, brain-gut hormones, and others. CRH, urotensin, urocortin, and stresscopin belong to the CRH family, and they all suppress feeding behavior (Furuse et al., 1997b; Zhang et al., 2001b; Cline et al., 2009b; Ogino et al., 2014). Melanocortin is derived from the precursor protein proopiomelanocortin (POMC). Among them, adrenocorticotropin hormone (ACTH) and α-melanocyte-stimulating hormone (α-MSH) have been demonstrated to suppress feeding behavior (Deviche and Delius, 1981; Kawakami et al., 2000a). In the glucagon superfamily, glucagon, glucagon-like peptide-1 (GLP-1), GLP-2, oxyntomodulin, vasoactive intestinal peptide (VIP), pituitary-adenylate cyclase-activating polypeptide (PACAP), and growth hormone-releasing hormone (GHRH) have been demonstrated to suppress feeding behavior (Furuse et al., 1997a; Tachibana et al., 2003a, 2015; Honda et al., 2007; Shousha et al., 2007; Cline et al., 2008b). Well-known brain-gut peptides that affect feeding behavior are CCK and bombesin-like peptides. In the bombesin-like peptides, gastrin-releasing peptide (GRP) and neuromedin B (both are homologs of bombesin) also suppress food intake after central injection (Tachibana et al., 2010b). Other peptides including vasotocin, mesotocin, neuromedin S, neuromedin U, neuropeptide FF, neuropeptide K, neuropeptide S, substance P, cocaine- and amphetamine-regulated transcript, and calcitonin gene-related peptide were reported to suppress feeding behavior (Tachibana et al., 2003b, 2004b, 2010a,c; Shousha et al., 2005b; Cline et al., 2007a,b, 2009a; Prall and Cline, 2008; Masunari et al., 2013). Most of the above are also known as anorexigenic peptides in mammalian species, although GHRH is thought to be an orexigenic neuropeptide in mammals.

**Table 1:** **Neuropeptides showing anorexigenic effects on feeding behavior in birds**.

**Anorexigenic neuropeptide**	**Change in food intake in neonatal chicks**
**CRH FAMILY**	
Corticotropin-releasing hormone (CRH)	Decreased
Urotensin	Decreased
Urocortin	Decreased
Stresscopin	Decreased
**MELANOCORTIN**	
Adrenocorticotropic hormone (ACTH)	Decreased
α-melanocyte-stimulating hormone (α-MSH)	Decreased
**GLUCAGON SUPERFAMILY**	
Glucagon	Decreased
Glucagon-like peptide-1 (GLP-1)	Decreased
Glucagon-like peptide-1 (GLP-2)	Decreased
Oxyntomodulin	Decreased
Vasoactive intestinal peptide (VIP)	Decreased
Pituitary-adenylate cyclase-activating polypeptide (PACAP)	Decreased
GCGL	Decreased
Growth hormone-releasing hormone (GHRH)	Decreased
**BRAIN-GUT PEPTIDES**	
Cholecystokinin (CCK)	Decreased
Gastrin	Decreased
Gastrin-releasing peptide (GRP)	Decreased
Neuromedin B	Decreased
Neuromedin C	Decreased
**OTHERS**	
Calcitonin gene-related peptide	Decreased
Cocaine-amphetamine regulated transcript	Decreased
Neuromedin S	Decreased
Neuromedin U	Decreased
Neuropeptide FF	Decreased
Neuropeptide K	Decreased
Neuropeptide S	Decreased
Mesotocin	Decreased
Substance P	Decreased
Vasotocin	Decreased

### CRH family

CRH is a 41-amino acid peptide and is well-known as a neuropeptide involved in the stress response (De Souza, 1995). CRH functions as the hypothalamic signal for the hypothalamus-pituitary-adrenal (HPA) axis and stimulates ACTH release from the anterior pituitary. There are two subtypes of CRH receptor, namely CRH-R1 and CRH-R2 (Hauger et al., 2003). The CRH receptors may also bind urotensins, which were originally isolated from the urophysis of teleosts. (Vaudry et al., 2010). Urotensin I is a paralog of CRH, and urotensin II shows structural similarity to somatostatin (Vaudry et al., 2010). Further work revealed that the *urotensin II* gene exists in mammals and birds (Vaudry et al., 2010). Urocortin is also a CRH-like peptide consisting of 40-amino acids and shows similarity to urotensin I (Hauger et al., 2003). Later, two isoforms of urocortin, urocortin-2, and urocortin-3, were identified (Hauger et al., 2003). At the same time, Hsu and Hsueh (2001) identified other CRH-like peptides, stresscopin and stresscopin-related peptide. Urocortin-3 and urocortin-2 are the C-terminus fragments of stresscopin and stresscopin-related peptide, respectively. While CRH binds to both CRH-R1 and R2, urocortin-2, urocortin-3, stresscopin-related peptide, and stresscopin are selective ligands for CRH-R2 (Hsu and Hsueh, 2001; Hauger et al., 2003).

ICV injection of CRH induces a stress-like response, such as hyperactivity, vocalization, hyperthermia, and increases in corticosterone release, in neonatal chicks (Zhang et al., 2001a; Tachibana et al., 2004b). As well as a stress-like response, CRH is also expected to be related to the inhibition of feeding behavior of neonatal chicks. In fact, ICV injection of CRH suppresses food intake (Furuse et al., 1997b). Furthermore, ICV injection of mammalian urotensin-1, urocortin-1, urocortin-3, and stresscopin decreases food intake in chicks (Zhang et al., 2001b; Cline et al., 2009b; Ogino et al., 2014). Thus, the CRH family peptides are expected to be anorexigenic neuropeptides in the brain of chicks. ICV injection of CRH increases the plasma corticosterone concentration in chicks, whereas the injection of urocortin-3 has no effect (Ogino et al., 2014), suggesting that urocortin-3 binds to a different receptor than CRH and exerts its effect. Because stresscopin and urcortin-3 show high affinity to the CRH-R2 receptor (Hsu and Hsueh, 2001; Hauger et al., 2003), CRH-R2 partly contributes to the anorexigenic effect of the CRH family peptides.

CRH has been demonstrated to modify the effect of other anorexigenic neuropeptides in chicks. Indeed, the anorexigenic effects of ghrelin, GLP-1, α-MSH, VIP, PACAP, glucagon, and CCK are partly attenuated by co-injection of CRH receptor antagonist (Tachibana et al., 2004c, 2006, 2007, 2012; Saito et al., 2005; Honda et al., 2012). Thus, it is possible that CRH might be one of the downstream mediators for the anorexigenic neural pathway in the brain of neonatal chicks.

### Melanocortins

Melanocortins, such as ACTH, α-MSH, β-MSH, and γ-MSH, are derived from the precursor POMC. To date, five melanocortin receptors (MC1R to MC5R) have been identified. In mammals, the melanocortin system plays an important role in regulating food intake and energy metabolism because disrupting melanocortin receptor-4 (MC4R) results in obesity, hyperphagia, and hyperglycemia in mice (Huszar et al., 1997). α-MSH is an endogenous MC4R agonist and decreases food intake in rats when injected centrally (Poggioli et al., 1986). Agouti-related protein (AGRP) is a naturally occurring antagonist for MC3R and MC4R (Fong et al., 1997), and its C-terminal fragment attenuates the anorexigenic effect of α-MSH in mammals (Rossi et al., 1998). Furthermore, the C-terminal fragment itself shows an orexigenic effect when administered centrally (Rossi et al., 1998). Thus, α-MSH and AGRP competitively regulate feeding behavior via MC4R in mammalian species.

The chicken *POMC* gene has been found to be a single copy gene and shows the same structural organization as in other vertebrates (Takeuchi et al., 1999). As in mammals, it has been demonstrated that ICV injection of α-MSH inhibits feeding behavior (Kawakami et al., 2000a), and the effect is attenuated by co-injection of AGRP in neonatal chicks (Tachibana et al., 2001a). Moreover, ICV injection of AGRP itself increases food intake in chicks and ring doves (Tachibana et al., 2001a; Strader et al., 2003). Notably, the orexigenic effect of AGRP depends on the strain of chicks: ICV injection of AGRP stimulates feeding behavior in layer chicks, whereas it has no effect on broiler chicks, which show rapid growth and heavy body weight compared with layer chicks (Tachibana et al., 2001a). Furthermore, a study using lines of White Plymouth Rock chickens reported that long-term divergent selection for low body weight shows high sensitivity to the anorexigenic effect of α-MSH, whereas sensitivity is low in the line selected for high body weight (Cline et al., 2008b). Thus, these results indicate that the melanocortin system is different between strains of chickens and is involved in regulating body weight in chicks.

In addition to alpha-MSH, the effect of some other melanocortins on feeding behavior has also been examined in birds. Although ACTH mainly exists in the anterior pituitary and functions as the pituitary signal of HPA axis, ACTH-containing neurons were found in several brain regions including the ARC of the hypothalamus (Csiffáry et al., 1990), and ACTH treatment suppresses feeding behavior in mammals (Al-Barazanji et al., 2001). The anorexigenic effect of ACTH was also observed in birds because ICV injection of ACTH suppresses feeding behavior in domestic pigeons (Deviche and Delius, 1981) and neonatal chicks (Shipp et al., 2015). On the other hand, chicken β-MSH and γ2-MSH have no effect on feeding in birds (Saneyasu et al., 2011).

### Glucagon superfamily

The glucagon superfamily, such as glucagon, GLP-1, GLP-2, VIP, PACAP, and GHRH, are also known to have an important role in regulating feeding behavior in birds (Honda, 2016). Glucagon, GLP-1, and GLP-2 are derived from the precursor protein proglucagon (Sherwood et al., 2000). These peptides are found in the peripheral tissue, but they are likely to exist in the central nervous system because proglucagon mRNA is expressed in the medulla oblongata of neonatal chicks (Tachibana et al., 2005a). In addition, food deprivation decreases the mRNA expression of proglucagon in the medulla oblongata, suggesting that endogenous proglucagon-derived peptides are related to the regulation of feeding behavior in neonatal chicks (Tachibana et al., 2005a). Indeed, ICV injection of GLP-1 has been demonstrated to decrease food intake in neonatal chicks (Furuse et al., 1997a) and Japanese quails (Shousha et al., 2007). In addition, ICV injection of GLP-1 induces Fos expression in the VMN in young chickens (Tachibana et al., 2004a), suggesting that central GLP-1 activates neurons in the VMN, and thereby suppresses feeding behavior. In fact, direct injection of GLP-1 into the VMN decreases food intake in young chickens. Not only exogenous GLP-1, but also endogenous GLP-1 is thought to be related to the inhibition of feeding behavior because ICV injection of the GLP-1 receptor antagonist exendin (5–39) stimulates feeding behavior in neonatal layer chicks (Tachibana et al., 2001b).

In addition to GLP-1, other proglucagon-derived peptides, such as glucagon, GLP-2, and oxyntomodulin, also suppress feeding behavior in neonatal chicks (Honda, 2016). Glucagon is a 29-amino acid peptide and is known as an important regulator of glucose metabolism (Sherwood et al., 2000). In chickens, glucagon receptor mRNA is expressed in the central nervous system, and is especially highly expressed in the hypothalamus in addition to several peripheral tissues (Wang et al., 2008). Although intravascular injection of glucagon has no effect on food intake, ICV injection of glucagon decreases food intake in neonatal chicks (Honda et al., 2007), suggesting that glucagon exerts its anorexigenic effect in the brain. Honda et al. (2015) also demonstrated that ICV injection of chicken GLP-2 decreases food intake in neonatal chicks, whereas human GLP-2 has no effect. Thus, central GLP-2 is expected to function as an anorexigenic neuropeptide in birds.

Oxyntomodulin shows the same amino acid sequence as glucagon at the N-terminus region followed by a 26-amino acid extension at its C-terminus in chickens (Honda, 2016). In mammals, the C-terminus amino acid extension is shorter than that in chickens (Honda, 2016), indicating that the structure of oxyntomodulin is different in chickens and mammals. Nevertheless, ICV injection of both mammalian and chicken oxyntomodulin decreases food intake in neonatal chicks (Cline et al., 2008a; Honda et al., 2014a). However, the anorexigenic effect of oxyntomodulin seems to be weaker than that of GLP-1 and GLP-2 (Honda et al., 2015). ICV injection of chicken oxyntomodulin increases plasma glucose and corticosterone levels in neonatal chicks (Honda et al., 2014a). Similar responses have also been observed after ICV injection of glucagon (Honda et al., 2007). In addition, the similarity of the amino acid sequence suggests that glucagon and oxyntomodulin suppress feeding behavior in chicks with the same neural networks. On the other hand, injection of GLP-2 decreases the plasma glucose level and has no effect on the plasma corticosterone level (Honda et al., 2015). In addition, injection of oxyntomodulin increases Fos expression in the IN and ARC, whereas it has no effect in the VMN in neonatal chicks (Cline et al., 2008a). Because GLP-1 induces Fos expression in the VMN (Tachibana et al., 2004a), it is likely that the anorexigenic neural pathway is different between oxyntomodulin, GLP-1, and GLP-2.

VIP and PACAP are also thought to suppress feeding behavior in chicks because ICV injections of these peptides decreases food intake in neonatal chicks (Tachibana et al., 2003a; Khan et al., 2013). ICV injection of anti-chicken VIP antiserum increases food intake in neonatal chicks, suggesting that central VIP is related to the inhibition of feeding. CRH is likely to mediate the feeding-inhibitory effect of VIP and PACAP because their anorexigenic effects are attenuated by co-injection of a CRH receptor antagonist in neonatal chicks (Tachibana et al., 2004c).

A novel glucagon-like peptide named GCGL is also thought to be related to the regulation of feeding behavior because ICV injection of GCGL decreases food intake in neonatal chicks (Honda et al., 2014b). The anorexigenic effect of GCGL is also mediated by the CRH system because co-injection of CRH receptor antagonist attenuates the effect of GCGL (Honda et al., 2014b). GCGL mRNA expression in the hypothalamus is not changed by 24-h food deprivation (Honda et al., 2014b), suggesting that central GCGL might not be related to normal feeding behavior but specific feeding, such as stress-related anorexia.

GHRH is recognized as a stimulator of GH release in mammals (Sherwood et al., 2000). In addition to the GH-releasing effect, GHRH is thought to be related to the regulation of feeding behavior because central injection of GHRH stimulates feeding behavior in rats (Vaccarino et al., 1985). In chickens, GHRH-like peptide (GHRH-LP) has been identified but its amino acid sequence shows low homology to mammalian GHRH (Sherwood et al., 2000). Furthermore, GHRH-LP is less potent in stimulating GH release in chickens (Harvey, 1999). These facts implied that there might be another GHRH in chickens. In 2007, Wang et al. (2007) identified chicken GHRH, which has higher affinity to chicken GHRH receptors than GHRH-LP (Wang et al., 2010). Although the amino acid sequence of this GHRH has low similarity to mammalian GHRH, a synteny analysis indicated that the chicken *GHRH* gene is located on a conserved synteny of all vertebrate species examined, including teleosts and amphibians (Wang et al., 2007). Based on these facts, it has been demonstrated that newly found GHRH is true GHRH in chickens. It is likely that GHRH and GHRH-LP are produced by the whole genome duplication (Wang et al., 2007).

ICV injection of synthesized chicken GHRH inhibits feeding behavior in neonatal chicks (Tachibana et al., 2015) as opposed to mammals. Notably, chicken GHRH-LP also suppresses feeding in chicks after ICV injection (Tachibana et al., 2015). Both GHRH and GHRH-LP have no effect on behavioral pattern and plasma corticosterone concentration, and it is likely that their anorexigenic effects may not be related to the induction of abnormal behavior, such as sleeping and hyperactivity, and to stress conditions (Tachibana et al., 2015). In addition, food deprivation affects mRNA expression of GHRH in the diencephalon (Tachibana et al., 2015), suggesting that endogenous GHRH in the brain is related to feeding regulation.

### Brain-gut peptides

CCK and gastrin are well-known as gastrointestinal hormones in vertebrates. Because these peptides share the same 5-amino acid sequence at the C-terminus, they belong to the same peptide family (Miyasaka and Funakoshi, 2003). CCK is present in a variety of biologically active peptides, such as CCK58, CCK33, and CCK8 derived from the precursor peptide (Miyasaka and Funakoshi, 2003). CCK has multiple effects on the gastrointestinal system including gallbladder contraction, gut motility, gastric emptying, and the secretion of gastric acid and pancreatic enzymes (Miyasaka and Funakoshi, 2003). Additionally, numerous studies have documented the satiety-inducing role of CCK in the brain. For example, central injection of CCK suppresses feeding behavior in sheep (Della-Fera and Baile, 1980).

CCK also inhibits feeding behavior in young chickens (Denbow and Myers, 1982) and neonatal chicks (Furuse et al., 2000; Tachibana et al., 2012) when administered centrally. The anorexigenic effect of CCK depends on the length of amino acids because ICV-injected CCK33S shows a stronger effect than CCK8S (Furuse et al., 2000), and CCK4 does not affect food intake in neonatal chicks (Tachibana et al., 2012). The effect of CCK is likely mediated by CRH because a CRH receptor antagonist attenuates the anorexigenic effect of CCK (Tachibana et al., 2012). In addition to CCK, ICV injection of gastrin decreases food intake in neonatal chicks (Furuse et al., 2000), suggesting that central gastrin is also related to the inhibition of feeding behavior.

Bombesin, a 14-amino acid peptide originally isolated from the skin of frog, suppresses feeding behavior when administered centrally and peripherally in young chickens (Denbow, 1989). In mammals and birds, there are two homologs of bombesin-like peptides called neuromedin B and gastrin-releasing peptide. The fragment of gastrin-releasing peptide is called neuromedin C. ICV injections of these bombesin-like peptides decreases food intake in neonatal chicks (Tachibana et al., 2010b), suggesting that they function as anorexigenic peptides in the brain of birds.

## Orexigenic neuropeptides

Table 2 summarizes the representative candidates of orexigenic neuropeptides in birds. Orexigenic peptides are roughly categorized as the families of pancreatic peptide, opioid and its related peptides, Arg-Phe-NH_2_ peptide (RFamide peptide), and others. In the pancreatic peptide family, NPY, pancreatic polypeptide (PP), and peptide YY (PYY) are thought to be orexigenic peptides in birds (Kuenzel et al., 1987; Ando et al., 2001). Opioid and its related peptides that stimulate feeding behavior in birds are β-endorphin, endomorphin-2, and nociception (Deviche and Schepers, 1984; Abbasnejad et al., 2005; Bungo et al., 2007). Gonadotropin-inhibiting hormone (GnIH), 26RFa, and prolactin-releasing peptide (PrRP), which are members of the RFamide peptide family, are also regarded as orexigenic peptides in birds (Tachibana et al., 2004d, 2005b; Ukena et al., 2010). In addition, AGRP, somatostatin, and galanin are also known as stimulators of feeding behavior in birds (Tachibana et al., 2001a, 2008b, 2009).

**Table 2:** **Neuropeptides showing orexigenic effects on feeding behavior in birds**.

**Orexigenic neuropeptide**	**Change in food intake in neonatal chicks**
**PANCREATIC PEPTIDE FAMILY**	
Neuropeptide Y (NPY)	Increased
Pancreatic peptide	Increased
Peptide YY(1–36)	Increased
**OPIOID AND ITS RELATED PEPTIDES**	
β-endorphin	Increased
Endomorphin-2	Increased
Nociception	Increased
**RFAMIDE PEPTIDES**	
Gonadotropin-inhibiting hormone (GnIH)	Increased
26RFa	Increased
Prolactin-releasing peptide (PrRP)	Increased
**OTHERS**	
Agouti-related protein (AGRP)	Increased
Galanin	Increased
Somatostatin	Increased

In mammals, melanin-concentrating hormone (MCH), motilin, and orexin are also known as orexigenic peptides (Garthwaite, 1985; Rossi et al., 1997; Sakurai et al., 1998). However, ICV injections of these peptides have no effect on food intake in neonatal chicks (Furuse et al., 1999; Ando et al., 2000). Ghrelin and GHRH are known as orexigenic neuropeptides in mammals. However, ghrelin and GHRH inhibit rather than stimulate feeding behavior in chicks (as noted above). Thus, it is likely that the feeding-stimulating neural networks in the brain of birds are different from that in other vertebrates.

### Pancreatic peptide family

NPY is a neuropeptide consisting of 36-amino acids and belongs to the pancreatic polypeptide family (Tatemoto et al., 1982). NPY is a potent orexigenic neuropeptide in mammals (Levine and Morley, 1984), reptiles (Morris and Crews, 1990), amphibians (Crespi et al., 2004), and teleosts (López-Patiño et al., 1999). Similarly, central injection of NPY stimulates feeding behavior in chickens (Kuenzel et al., 1987; Chen et al., 2016), white-crowned sparrows (Richardson et al., 1995), and ring doves (Strader and Buntin, 2001). ICV injection of chicken NPY increases food intake and anti-chicken NPY antibody decreases food intake in neonatal chicks (Chen et al., 2016). In addition, fasting increases NPY content in the PVN and IN of the hypothalamus in young chickens (Zhou et al., 2005). These results suggest that endogenous NPY in the brain may function as an orexigenic neuropeptide in chicks. Furthermore, the NPY content in the hypothalamus of embryos and the mRNA expression level of NPY in the hypothalamic nuclei in chickens are different between layers and broilers (Zhou et al., 2006; Chen et al., 2007). It is therefore possible that NPY contributes to the difference in food intake and growth rate in chickens.

NPY-containing neurons in the IN of the hypothalamus are co-localized with the insulin receptor in neonatal chicks (Shiraishi et al., 2011). In addition, NPY mRNA in the brainstem is downregulated by insulin (Shiraishi et al., 2008). These findings suggest that the activity of the NPY neuron is regulated by insulin. In addition, other neuropeptides regulate the mRNA expression of NPY. For example, ICV injection of GnIH upregulates NPY mRNA expression in the hypothalamus (McConn et al., 2014). As well as the potent orexigenic effect, the existence of neural networks with other feeding regulatory peptides implies that NPY plays a key role in regulating feeding behavior in chicks.

PP is also expected to possess an orexigenic effect in chickens because ICV injection of avian PP increases food intake in young chickens (Kuenzel et al., 1987). A similar effect was also reported in neonatal chicks: ICV injection of human or rat PP increases food intake (Ando et al., 2001). Because centrally-injected PP also stimulates feeding behavior in mice (Asakawa et al., 1999), it is likely that the role of PP in the brain is conserved between chicks and rodents.

PYY belongs to the pancreatic family and is released from the gastrointestinal tract. There are two major forms of PYY, namely PYY(1-36) and PYY(3-36). ICV injection of PYY(1-36) stimulates feeding behavior in rats (Clark et al., 1987). In contrast, IP injection of PYY(3-36) inhibits feeding behavior (Batterham et al., 2002). PYY(3-36) binds to the NPY Y2 receptor, which is highly expressed on NPY neurons in the ARC of the hypothalamus and suppresses the activity of NPY neurons (Batterham et al., 2002). Intra-ARC injection of PYY(3–36) suppresses feeding behavior (Batterham et al., 2002), demonstrating that PYY(3–36) is an anorexigenic neuropeptide as a peripheral factor in mammals. In neonatal chicks, ICV injection of mammalian PYY(1–36) increases food intake as it does in rats (Ando et al., 2001). Recently, Aoki et al. (2016) identified cDNA of chicken PYY precursor and found that intravenous injection of PYY(3–36) decreases food intake in neonatal chicks. This result indicates that peripheral PYY(3–36) functions as an anorexigenic neuropeptide in chickens.

### Opioid and its related peptides

In mammals, the opioid system in the central nervous system was reported to stimulate feeding behavior (Kuenzel, 1994). Similarly, ICV injection of β-endorphin, an endogenous opioid, stimulates feeding behavior in pigeons (Deviche and Schepers, 1984), young chickens (McCormack and Denbow, 1988), and white-crowned sparrows (Maney and Wingfield, 1998). These results indicate that the central opioid system functions as an orexigenic neuropeptide in birds.

Among opioid receptors, δ- and κ-receptors are thought to be related to the orexigenic effect of opioids because ICV injection of δ-receptor agonists ([D-Ala^2^, D-Leu^3^]-enkephalin and [D-Pen^2, 5^]-enkephalin) and κ-receptor agonists (U-50488H and U-62066) increase food intake in chicks (Bungo et al., 2004). On the other hand, a μ-receptor agonist, [D-Ala^2^, N-MePhe4, Gly^5^-ol]-enkephalin, decreases food intake in neonatal chicks probably because of the sleep-like behavior induced by this agonist (Bungo et al., 2004). McCormack and Denbow (1987) reported that intramuscular injection of naloxone, an opioid μ-receptor antagonist, suppresses feeding behavior in young chickens. Furthermore, ICV injection of naloxone suppresses feeding behavior in white-crowned sparrows (Maney and Wingfield, 1998). Bungo et al. (2005) demonstrated that ICV injection of the μ-receptor antagonist, β-funaltrexamine, decreases food intake in neonatal chicks. Based on the studies using antagonists for the μ-receptor, this receptor is also expected to be related to the orexigenic effect of opioid in birds. In fact, the μ-receptor is demonstrated to mediate the orexigenic effect of other orexigenic peptides, such as NPY (Dodo et al., 2005), GnIH (Tachibana et al., 2008a), galanin (Tachibana et al., 2008b), and somatostatin (Tachibana et al., 2009). The opioid system is likely a downstream mediator for the orexigenic neural networks in the brain of birds.

Nociceptin (orphanin FQ), a 17-amino acid peptide, is an endogenous ligand of the nociceptin receptor, which shows structural similarity to the opioid receptor. Nociceptin is thought to have an orexigenic effect in mammals (Stratford et al., 1997). The orexigenic effect of nociceptin is also observed in chickens: ICV injection of nociceptin increases food intake and feeding time (Abbasnejad et al., 2005). Thus, the role of the opioid system in feeding behavior seems to be conserved between birds and mammals.

### RFamide peptides

GnIH is a dodecapeptide possessing a C-terminal sequence, Arg-Phe-NH_2_ (RFamide peptide) (Tsutsui et al., 2000), and suppress gonadotropin release in birds and other vertebrates (Tsutsui, 2009; Tsutsui et al., 2010). Because GnIH inhibits gonadotropin release in quail and other birds, this peptide is an important factor in regulating avian reproduction (Tsutsui, 2009; Tsutsui et al., 2010). The distribution of GnIH and its receptor in the brain (Bentley et al., 2003; Ubuka et al., 2003; Yin et al., 2005) indicates that GnIH is not only related to the reproduction but also to behavioral and autonomic mechanisms (Tsutsui, 2009; Tsutsui et al., 2010). It has been demonstrated that food restriction decreases the release of gonadotropin and sex steroids in domestic hens (Richard-Yris et al., 1987). This finding suggests that gonadotropin and sex steroids are related to the regulation of energy homeostasis, including feeding behavior in avian species. In fact, ICV injection of quail and chicken GnIH and its related peptides increases food intake in neonatal chicks (Tachibana et al., 2005b; McConn et al., 2014). A similar effect of GnIH is also observed in Pekin duck (Fraley et al., 2013). ICV injection of anti-GnIH antiserum decreases deprivation-induced feeding in neonatal chicks (Tachibana et al., 2005b). Food deprivation for 48-h induces Fos expression in GnIH-immunoreactive neurons in the PVN of the hypothalamus of Pekin duck (Fraley et al., 2013). Moreover, GnIH mRNA expression increases in the hypothalamus of neonatal chicks by fasting (McConn et al., 2016). These results suggest that endogenous GnIH in the brain is related to the regulation of feeding behavior in birds. It is likely that the orexigenic effect of GnIH is mediated by the opioid and NPY systems in neonatal chicks (Tachibana et al., 2008a; McConn et al., 2014).

26RFa was originally isolated from frog brains (Chartrel et al., 2003). This peptide was named based on its features: It consists of a 26-amino acid residue and possesses an RFamide sequence at its C-terminus. 26RFa is also found in humans and rats, and its mRNA is distributed in the LHA and VMN of the hypothalamus of rats (Chartrel et al., 2003). ICV injection of 26RFa stimulates feeding behavior in mice (Chartrel et al., 2003), suggesting that this peptide functions as an orexigenic neuropeptide in mammals. 26RFa was also identified in birds including quail and chickens (Ukena et al., 2010). The 26RFa mRNA is expressed in the diencephalon that includes the hypothalamus, and 26RFa-containing perikarya were found in the anterior hypothalamic nucleus in quail and chicks (Ukena et al., 2010). In addition, the mRNA of GPR103, a receptor for 26RFa, is distributed more in the diencephalon than other brain regions (Ukena et al., 2010). These findings suggest that 26RFa is involved in regulating feeding behavior in birds. Indeed, ICV injection of 26RFa increases food intake in neonatal broiler chicks, although it has no effect in layer chicks (Ukena et al., 2010).

PrRP was first isolated from the hypothalamus as a specific prolactin-releasing factor for mammalian pituitary cells (Hinuma et al., 1998). However, subsequent studies revealed that the peptide has less effect on prolactin release in mammals (Maruyama et al., 1999). On the other hand, ICV injection of PrRP has been demonstrated to suppress feeding behavior in rats (Lawrence et al., 2000). Concurrently with the discovery of PrRP, Carassius Arg-Phe-NH_2_ peptide (C-RFa), an ortholog of PrRP, was isolated from Japanese crucian carp (Fujimoto et al., 1998). Central and peripheral injection of fish PrRP inhibits feeding behavior in goldfish (Kelly and Peter, 2006), suggesting that PrRP functions as an anorexigenic peptide in mammals and teleosts. However, in neonatal chicks ICV injection of mammalian PrRP31 stimulates rather than inhibits feeding behavior (Tachibana et al., 2004d). After the discovery of chicken PrRP, the orexigenic effect of PrRP was confirmed: ICV injection of chicken PrRP increases food intake in neonatal chicks (Tachibana et al., 2011). These results suggest that PrRP might be an orexigenic neuropeptide in birds, unlike in mammals and teleosts. Wang et al. (2012) identified new chicken PrRP and showed that there are two types of PrRP encoded by separate genes. They also demonstrated that the chicken PrRP we previously identified (Tachibana et al., 2011; Tachibana and Sakamoto, 2014) is an ortholog of C-RFa, and newly identified chicken PrRP is an ortholog of mammalian PrRP. These two types of PrRP are thought to be produced by whole genome duplication (Wang et al., 2012; Tachibana and Sakamoto, 2014). Since newly identified chicken PrRP also increases food intake in neonatal chicks when administered centrally (Tachibana and Sakamoto, 2014), the role of PrRPs on feeding behavior is expected to have changed during the evolution of vertebrates.

### Somatostatin

Somatostatin is well-known as a hypothalamic inhibitor of GH release from the anterior pituitary (Brazeau et al., 1973). This neuropeptide also affects feeding behavior in mammals because ICV injection of somatostatin suppresses feeding behavior in chicks (Vijayan and McCann, 1977). However, the effect of somatostatin on feeding behavior depends on the experimental condition (Feifel and Vaccarino, 1990). Somatostatin also has an inhibitory effect on GH release in chickens (Harvey and Scanes, 1987). Although somatostatin affects feeding behavior in chicks as well as mammals, it consistently stimulates feeding behavior in neonatal chicks after ICV injection (Tachibana et al., 2009). The injection of coristatin, a neuropeptide structurally related to somatostatin, also stimulates feeding behavior in neonatal chicks (Tachibana et al., 2009).

As noted above, it is likely that the effects of GH-related peptides including ghrelin and GHRH are different between chicks and mammals. In mammals, peptides that stimulate GH release usually show an orexigenic effect, whereas peptides that inhibit GH release partly have an anorexigenic effect. In chicks, on the other hand, GHRH and ghrelin possess an anorexigenic effect, whereas somatostatin possesses an orexigenic effect. Because most of these studies were performed by injecting exogenous peptides, further studies on the effect of endogenous GH-related peptides should show the actual relationship between these peptides and feeding behavior in chicks.

### Galanin

Galanin is a neuropeptide consisting of 29-amino acids and is distributed in the brain and digestive tract. Central injection of galanin stimulates feeding behavior in rats (Kyrkouli et al., 1986) and goldfish (de Pedro et al., 1995). Similarly, ICV injection of mammalian galanin increases food intake in neonatal chicks (Tachibana et al., 2008b). These results suggest that the orexigenic effect of galanin is conserved in vertebrates.

## Conclusion

As noted above, several kinds of peptidergic molecules are related to the regulation of feeding behavior in birds, and some neuropeptides show different effects from that in other vertebrates. Notably, non-peptidergic feeding regulatory factors, such as norepinephrine, 5-hydroxytryptamine, and histamine, show a similar effect in vertebrates when administered centrally (Denbow et al., 1982; Bungo et al., 1999a; Kawakami et al., 2000b). Why avian species have acquired distinct effects in response to feeding regulatory peptides compared to mammals is still unknown. The most marked trait in birds is adaptation for flying. For the purpose of flying, birds possess a toothless beak, feathers and wings, a unique digestive tract and respiratory system, and a high metabolic rate. Their short rectum and lightweight skeleton are also adaptations for flying. Flying requires more energy but overeating disturbs flying. This might be a partial reason why the effect of orexigenic neuropeptides in birds is different than in other vertebrates.

However, most of the studies on feeding regulation in birds have used exogenous peptide treatments. Studies on the roles of endogenous feeding regulatory peptides are needed to understand the true physiological regulation of feeding behavior in birds. In addition, it should be noted that most of the studies used neonatal and young chickens. It is possible that the roles of neuropeptides in regulating feeding behavior vary with age. Studies using adult birds should clarify the actual difference in neuropeptide control of feeding behavior between vertebrates. Clarifying the feeding regulatory mechanism should provide information on the evolution of feeding regulation in vertebrates and would be beneficial information for poultry production.

## Author contributions

TT wrote this review together with co-author KT, Waseda University, Japan. In addition, TT integrated KT's sentences and edited this review as the corresponding author.

### Conflict of interest statement

The authors declare that the research was conducted in the absence of any commercial or financial relationships that could be construed as a potential conflict of interest.
